# Early age at first childbirth and skilled birth attendance during delivery among young women in sub-Saharan Africa

**DOI:** 10.1186/s12884-021-04280-9

**Published:** 2021-12-14

**Authors:** Eugene Budu, Vijay Kumar Chattu, Bright Opoku Ahinkorah, Abdul-Aziz Seidu, Aliu Mohammed, Justice Kanor Tetteh, Francis Arthur-Holmes, Collins Adu, Sanni Yaya

**Affiliations:** 1 Department of Population and Health, University of Cape Coast, Cape Coast, Ghana; 2 Department of Public Health, Saveetha Medical College and Hospitals, SIMATS, Saveetha University, Chennai, TN 600077 India; 3 Department of Community Medicine, Faculty of Medicine, Datta Meghe Institute of Medical Sciences (Deemed University), Wardha, MS 442107 India; 4 School of Public Health, Faculty of Health, University of Technology Sydney, Sydney, Australia; 5 College of Public Health, Medical and Veterinary Sciences, James Cook University, Townsville, Australia; 6 Centre For Gender and Advocacy, Takoradi Technical University, P.O.Box 256, Takoradi, Ghana; 7 Department of Health, Physical Education and Recreation, University of Cape Coast, Cape Coast, Ghana; 8 Department of Sociology and Social Policy, Lingnan University, 8 Castle Peak Road, Tuen Mun, Hong Kong; 9 Department of Health Promotion, Education and Disability Studies, Kwame Nkrumah University of Science and Technology, Kumasi, Ghana; 10 School of International Development and Global Studies, University of Ottawa, Ottawa, Canada; 11 The George Institute for Global Health, Imperial College London, London, UK

**Keywords:** Age at first birth, Skilled birth attendance, Young women, Sub-Saharan Africa, Global health, Demographic and health surveys

## Abstract

**Background:**

Despite the numerous policy interventions targeted at preventing early age at first childbirth globally, the prevalence of adolescent childbirth remains high. Meanwhile, skilled birth attendance is considered essential in preventing childbirth-related complications and deaths among adolescent mothers. Therefore, we estimated the prevalence of early age at first childbirth and skilled birth attendance among young women in sub-Saharan Africa and investigated the association between them.

**Methods:**

Demographic and Health Survey data of 29 sub-Saharan African countries was utilized. Skilled birth attendance and age at first birth were the outcome and the key explanatory variables in this study respectively. Overall, a total of 52,875 young women aged 20-24 years were included in our study. A multilevel binary logistic regression analysis was performed and the results presented as crude and adjusted odds ratios at 95% confidence interval.

**Results:**

Approximately 73% of young women had their first birth when they were less than 20 years with Chad having the highest proportion (85.7%) and Rwanda recording the lowest (43.3%). The average proportion of those who had skilled assistance during delivery in the 29 sub-Saharan African countries was 75.3% and this ranged from 38.4% in Chad to 93.7% in Rwanda. Young women who had their first birth at the age of 20-24 were more likely to have skilled birth attendance during delivery (aOR = 2.4, CI = 2.24-2.53) than those who had their first birth before 20 years.

**Conclusion:**

Early age at first childbirth has been found to be associated with low skilled assistance during delivery. These findings re-emphasize the need for sub-Saharan African countries to implement programs that will sensitize and encourage the patronage of skilled birth attendance among young women in order to reduce complications and maternal mortalities. The lower likelihood of skilled birth attendance among young women who had their first birth when they were adolescents could mean that this cohort of young women face some barriers in accessing maternal healthcare services.

## Background

Despite the numerous policy interventions aimed at preventing adolescent childbearing in many countries across the world, the prevalence of adolescent pregnancy and childbirth remains high [1–3]. Adolescent childbearing has been defined as birth that occurs among adolescents aged 10-19 [4]. In 2015, for example, approximately 19.4 million adolescent girls aged 10-19 years, gave birth, and 580,000 of them were aged 10-14 [4]. Most of these adolescent births occur in less developed countries, especially in sub-Saharan Africa (SSA) [5, 6]. Besides, childbearing related complications and delivery are reported to be the major cause of mortality among adolescent girls (aged 15-19) in the world [2, 3].

In SSA, an estimated 570 adolescent girls die each year due to maternal complications compared to 22 in Europe, 61 in the Americas, 77 in the Western Pacific, 130 in Southeast Asia, and 430 in the Eastern Mediterranean [7]. Again, children born to adolescent mothers have a higher risk of health complications and mortality than those born to older women [5, 8]. It is also reported that adolescent girls have higher odds of complicated pregnancy outcomes than older women [5]. In relation to this, Grønvik and FossgardSandøy [8] reported that adolescent girls in SSA have a higher risk for prenatal and maternal mortality, low birth weight, eclampsia and preterm delivery. Other complications associated with adolescent childbearing include haemorrhage [4], systemic infections, puerperal endometritis, and increased risk for caesarean sections [7, 9].

Available evidence suggests that skilled birth attendance (SBA) is an important maternal health service that reduces adverse pregnancy outcomes among childbearing women and minimises post-delivery complications [4, 10]. Therefore, increasing the proportion of skilled birth attendance is one of the surest ways of ending preventable maternal deaths in SSA and reducing the global maternal mortality rate to less than 70 per 100,000 live births by 2030 [SDG 3.1] [4, 11]. However, the utilization of skilled deliveries remains relatively low among adolescent mothers in most sub-Saharan African countries [12, 13] Mekonnen et al. [14] estimated that the prevalence of skilled delivery among adolescent mothers in SSA ranged from 10% in Ethiopia to 72% in Guinea.

Previous studies have reported that skilled birth delivery in SSA is associated with factors such as age, parity, wealth quintile [13], level of education, antenatal care attendance, access to electronic media [15], and rural/urban residence [15, 16]. Other factors include the distance from the health facility, male involvement, and mother’s knowledge of pregnancy risk factors [17]. In Ghana, for example, Nuamah et al. [13] reported that older mothers (> 34 years) had higher odds of SBA during delivery than younger women (< 24 years). In Ethiopia, women who have access to television or those attending antenatal care at least 4 times have higher odds of SBA [15]. Similar studies conducted previously in SSA were mostly conducted among women aged 15-49 years and did not specifically focus on adolescent girls [13, 15, 17]. However, few studies that focused on adolescent childbearing mostly investigated maternal services utilization such as SBA, antenatal care, and postnatal care [14, 18, 19].

Considering the negative effects of early childbearing on maternal healthcare services utilization [14, 20, 21], it is important to understand how early age at first birth affects the utilization of SBA among young women in SSA, especially because early childbearing could have long term effect on SBA use. Therefore, we estimated the prevalence of early age at first childbirth and skilled birth attendance among young women in sub-Saharan Africa and. We also investigated the relationship between them. This study’s findings will help stakeholders including health authorities to develop interventions and health programs to improve SBA among pregnant adolescent in SSA.

## Methods

### Data source

Demographic and Health Surveys (DHS) data of 29 sub-Saharan African countries were used for the study (Table 1). Specifically, data from the birth recode files were considered. The DHS is conducted in over 85 low- and middle-income countries and they are nationally representative. The DHS looks at important markers such as SBA [22]. A two-stage stratified sampling technique is used for the nationwide survey and this makes the data representative of each country. The sampling procedure employed for the surveys have been well documented in literature [23]. Young women (aged 20-24) totaling 52,875 with complete information on all the variables of interest were included in our study. Strengthening the Reporting of Observational Studies in Epidemiology’ (STROBE) statement was used as a guide to help in writing the manuscript [24]. The dataset is available and free for download at https://dhsprogram.com/data/available-datasets.cfm

**Table 1:** Description of the study sample

Countries	Year of survey	Women aged 20-24 years	Women with a birth history	Women with complete cases
1. Angola	2015-16	2988	2323	2323
2. Burkina Faso	2018	3267	2529	2528
3. Benin	2017-18	2880	1986	1985
4. Burundi	2016-17	3210	1805	1805
5. Congo DR	2018	3649	2608	2601
6. Congo	2014-15	1983	1472	1472
7. Cote d’Ivoire	2012	1924	1310	1304
8. Cameroon	2011-12	3283	1550	1549
9. Ethiopia	2013-14	2728	1543	1543
10. Gabon	2011-12	1599	1020	1020
11. Ghana	2016	1591	773	773
12. Gambia	2012	2099	1202	1202
13. Guinea	2013	1729	1167	1167
14. Kenya	2014	5662	3747	3742
15. Comoros	2018	968	391	391
16. Liberia	2014	1615	1316	1316
17. Lesotho	2014	1306	817	818
18. Mali	2013	1871	1496	1496
19. Malawi	2015-16	5083	4091	4091
20. Nigeria	2018	6749	4351	4351
21. Namibia	2013	1761	1010	1009
22. Rwanda	2018	2427	1180	1180
23. Sierra Leone	2014-15	2629	1945	1945
24. Senegal	2010-11	3174	1739	1739
25. Chad	2013	3016	2517	2503
26. Togo	2013-14	1640	922	922
27. Uganda	2016	3765	2850	2850
28. Zambia	2018	2700	2040	2040
29. Zimbabwe	2015	1674	1204	1204
**Total**		**78,063**	**52,906**	**52,875**

### Definition of variables

#### Outcome variable

This study used assistance during delivery as the main outcome variable. Assistance during delivery was obtained from the question, “Who assisted [NAME] during delivery?”. Responses to the question was categorized into “Traditional Birth Attendants/Others (traditional health volunteer, community/village health volunteer, neighbors/ friends/relatives and other people and “skilled birth attendants (doctor, nurse, auxiliary midwife, or nurse/midwife).

#### Independent variables

The study’s primary explanatory variable was “age at first birth,” which was obtained from the question, “how old were you when you first gave birth?”. For this study, the responses were re-coded into “early age at first birth” = 1 and “late age at first birth” = 2, where “early age at first birth” and “late age at first birth” represented the respondents who gave birth between age 10-19 and age 20-24 respectively.

#### Control variables

Five individual and five contextual level variables were the focus of this study. The individual-level variables comprised education (no education, primary and secondary/higher), marital status (not married, married, cohabiting, widowed, divorced/separated), parity (one birth, two births, three births and four or more births), mass media exposure included exposure to the newspaper, radio and television (no and yes), and religion (Christianity, Islam and others). The contextual level variables were wealth index (poorest, poorer, middle, richer and richest), sex of household head (male and female), community literacy level – the proportion of women in the community who can read and write (low, middle and high), community socio-economic status – the proportion of women in the community with richest wealth quintile (low, medium and high), place of residence (urban and rural) and sub-region (West Africa, East Africa, Central Africa and Southern Africa). The sub-Saharan African countries included in this study were Ghana, Mali, Burkina Faso, Cote d’Ivoire, Benin, Senegal, Zimbabwe, Gambia, Namibia, Guinea, Nigeria, Gabon, Sierra Leone, Togo, Burundi, Cameroon, Uganda, Ethiopia, Kenya, Comoros, Malawi, Rwanda, Zambia, Angola, Congo DR, Congo, Liberia, Chad, and Lesotho [25]. The variables of this study were derived with respect to their theoretical relevance, parsimony and practical significance with SBA during delivery [20, 26–29].

#### Statistical analyses

**Fig. 1:**
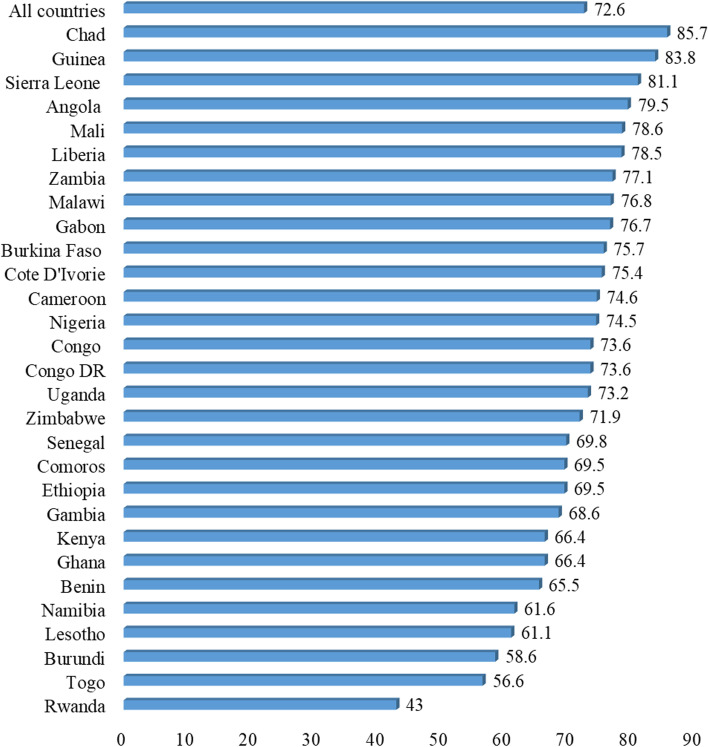
Proportion of young women whose first childbirth occurred when they were adolescents in sub-Saharan Africa

**Fig. 2:**
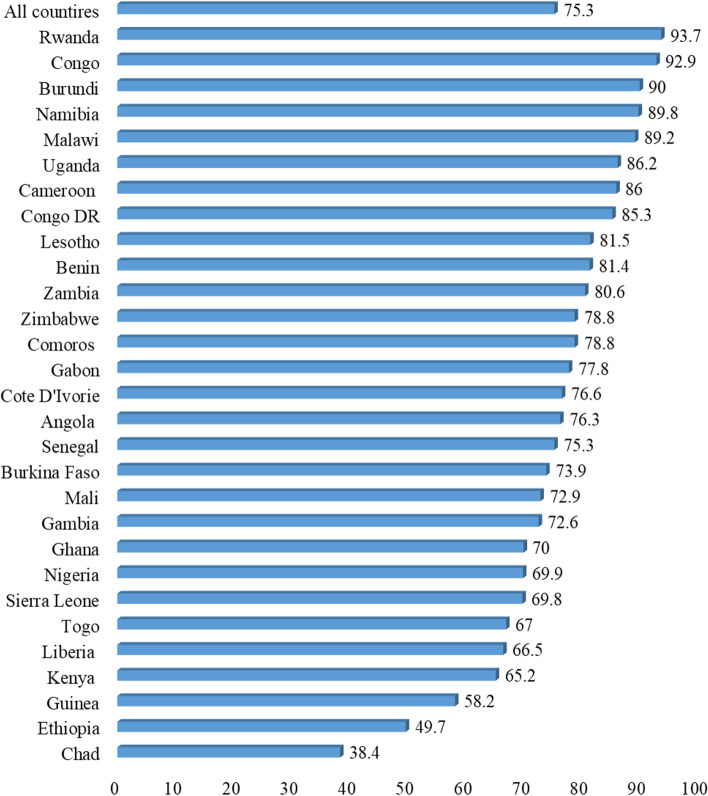
Proportion of young women who had skilled assistance during delivery in sub-Saharan Africa

The data analysis was executed with Stata version 14.0. The analysis was  done in three phases. The first phase comprised the calculation of the prevalence of SBA (Fig. 1) and early age at first childbirth (Fig. 2). The second phase involved a bivariate analysis that estimated SBA prevalence across the independent and control variables with their significance levels (Table 2). Using the variance inflation factor (VIF), a test for multicollinearity was then carried out and the results showed no evidence of high collinearity (Mean VIF = 1.49, Maximum VIF = 2.46, and Minimum VIF = 1.02). The test for collinearity was conducted to check for a high correlation among the explanatory variables. From Table 2, all variables that showed statistical significance were included in a two-level multilevel logistic regression analysis that had five models. The first model (Model O) was the empty model that showed the variance in SBA in the absence of the explanatory variables. Model I had only age at first birth and SBA. Model II contained the individual-level variables and SBA. Model III had the contextual level variables and SBA. The final model (Model IV) contained age at first birth, the control variables and SBA . The multilevel logistic regression analysis comprised fixed and random effects [30]. The purpose of different models was due to the nature of the control variables which were grouped into individual and contextual variables. We wanted to see how the inclusion of each set of variable would affect the relationship between age at first birth andSBA. In this study, fixed effects results of the model were presented as crude odds ratio (cOR) and adjusted odds ratio (aOR) whiles the random effects were examined using Intra-Cluster Correlation (ICC) [30]. The log-likelihood ratio (LLR) and Akaike’s Information Criterion (AIC) tests were used for the model comparisons. In Stata, during the regression analysis, we employed the survey command (svy) to adjust for the complex sampling structure of the data. We also weighted all frequency distributions. Since this was a pooled data, the survey weight in each country’s dataset was de-normalized and re-normalized based on the population sizes of the countries in the study and the new weights generated were used in the appended dataset for the analysis.

**Table 2:** Distribution of skilled birth attendance during delivery by age at first birth and individual and contextual characteristics of young women in sub-Saharan Africa

Variables	Weighted N	Weighted %	Skilled birth attendance during delivery	***p***-value
**Age at first birth**				< 0.001
< 20 years	38,380	72.6	70.9	
20-24 years	14,495	27.4	87.1	
**Level of education**				< 0.001
No education	15,524	29.4	61.5	
Primary	18,242	34.5	77.3	
Secondary +	19,109	36.1	84.7	
**Marital status**				< 0.001
Not married	7388	14.0	79.3	
Married	31,805	60.2	72.9	
Cohabiting	9627	18.2	80.6	
Widowed	286	0.5	65.1	
Divorced/separated	3769	7.1	75.4	
**Parity**				< 0.001
One birth	23,989	45.4	79.3	
Two births	17,940	33.9	75.1	
Three births	7992	15.1	68.9	
Four or more births	2954	5.6	62.3	
**Wealth index**				< 0.001
Poorest	10,806	20.4	64.0	
Poorer	11,619	22.0	71.1	
Middle	10,788	20.4	75.9	
Richer	10,749	20.3	81.3	
Richest	8913	16.9	86.6	
**Mass media exposure**				< 0.001
No	19,341	36.6	69.6	
Yes	33,534	63.4	78.7	
**Sex of head of household**				0.003
Male	40,940	77.4	74.9	
Female	11,935	22.6	76.8	
**Religion**				< 0.001
Christianity	33,242	62.9	80.0	
Islam	17,452	33.0	67.5	
Others	2181	4.1	67.6	
**Residence**				< 0.001
Urban	18,392	34.8	83.7	
Rural	34,483	65.2	70.9	
**Community-level literacy**				< 0.001
Low	18,992	35.9	66.3	
Medium	17,948	33.9	77.3	
High	15,935	30.2	83.9	
**Community socio-economic level**				< 0.001
Low	26,349	49.8	67.6	
Medium	10,142	19.2	80.0	
High	16,383	31.0	84.8	
**Sub-region**				< 0.001
West Africa	19,417	36.7	72.1	
East Africa	21,416	40.5	79.7	
Central Africa	10,215	19.3	70.5	
Southern Africa	1827	3.5	86.1	

## Results

### Proportion of young women whose first birth occurred when they were adolescents and those who had skilled assistance during delivery in sub-Saharan Africa

Figure 1 shows the proportions of young women whose first birth occurred during adolescence in the 29 sub-Saharan African countries. The overall proportion of young women whose first birth occurred during adolescence was 72.6%. Rwanda (43.3%) recorded the lowest proportion of young women whose first birth occurred during adolescence, with Chad having the highest proportion (85.7%). The majority of the countries recorded 70-80% of young women having their first birth before 20 years.

The average proportion of skilled assistance during delivery in SSA was 75.3%, ranging from 38.4% in Chad to 93.7% in Rwanda. It is important to mention that countries like Congo (92.9%), Burundi (90%), Namibia (89.8%) and Malawi (89.2%) also recorded higher skilled assistance during delivery (see Fig. 2).

### Distribution of skilled birth attendance during delivery across age at first birth and individual and contextual characteristics of young women in sub-Saharan Africa

Table 2 presents the distribution of SBA during delivery across age at first birth and socio-demographic characteristics of young women in SSA. Women whose first birth occurred at age 20-24 had a higher prevalence of skilled assistance during delivery (87.1%) than those whose first birth occurred before 20 years (70.9%). Women in the primary (77.3%) and secondary/higher education category (84.7%) had a higher prevalence of skilled assistance during delivery than those without formal education. Cohabiting women (80.9%) had the highest prevalence of skilled birth assistance  in terms of marital status. Women with one parity (79.3%) had a higher prevalence than those with four or more births (62.3%). Also, skilled assistance during delivery was more prevalent among women in the richest (86.6%) and richer (81.3%) wealth quintile than those in the poorer (71.1%) and poorest (64.0%) wealth quintile. However, women in both male (74.9%) and female (76.8%) headed households had a higher prevalence of skilled assistance during delivery with a difference of 1.9%. Skilled assistance during delivery was more prevalent among urban residents (83.7%) than rural residents (70.9%). Skilled assistance during delivery was higher among Christians (80.0%) than Muslims (67.5%) and women of other religions (67.6%). Lastly, women with high community-level literacy (83.9%) and socioeconomic status (84.8%) had a higher prevalence of skilled assistance during delivery than those with low and medium community level literacy and socio-economic status (Table 2).

### Fixed and random effects of results on the association between early age at first birth and skilled birth attendance among young women

Table 3 shows the fixed and random effects of the association between early age at first birth and SBA among young women. In terms of the random effects results, the clustering of the primary sampling units (PSUs) in “model O[Null model]” was responsible for significant differences in the odds of SBA (σ2 = 0.14, 95% CI 0.11-0.17). Model O showed that 4% of the total variation in SBA was attributed to the variance between clusters (ICC = 0.04). The between-cluster variance remained the same (ICC = 0.04) in Model I, rounding off to 2 decimal places . From Model I, the ICC increased to 6% in Model II but decreased to 4% in Model III. It then increased to 6% in Model IV, where all the independent variables (both individual and community level variables) were considered. This indicates that differences in the PSUs’ clustering account for the variations in SBA. The highest log-likelihood (− 27,255.173) and the lowest AIC (54,560.35) were used to determine the best fit model (See Table 3). The fixed results of the analysis are also shown in Table 3. In Model I, women who gave birth at the age of 20-24 were 2.7 times more likely to have SBA during delivery than those with first birth before 20 years (OR = 2.69, CI = 2.55-2.84). After controlling for all the individual and community level factors in Model IV, women whose first birth occurred at the age of 20-24 still had higher odds of SBA during delivery (aOR = 2.37, CI = 2.23-2.5) than those with first birth before 20 years. Level of education, marital status, parity, religion, wealth index, sex of household head, community literacy level, community socio-economic status, and place of residence showed statistically significant associations with SBA during delivery (Table 3).

**Table 3:** Fixed and random effects results on the association between adolescent childbearing and skilled birth attendance

Variables	Null model	Model I AOR[95%CI]	Model II AOR[95%CI]	Model III AOR[95%CI]	Model IV AOR[95%CI]
**Age at first birth**					
< 20 years		1			1
20-24 years		2.69^***^ (2.55-2.84)			2.37^***^ (2.23-2.52)
**Level of education**					
No education			0.37^***^ (0.34-0.39)		0.50^***^ (0.47-0.54)
Primary			0.67^***^ (0.63-0.70)		0.85^***^ (0.80-0.90)
Secondary +			1		1
**Marital status**					
Not married			0.85^***^ (0.79-0.91)		0.91^**^ (0.84-0.98)
Married			1		1
Cohabiting			1.17^***^ (1.10-1.25)		1.31^***^ (1.22-1.40)
Widowed			0.64^***^ (0.49-0.82)		0.68^**^ (0.52-0.88)
Divorced/separated			0.82^***^ (0.75-0.89)		0.91^*^ (0.83-0.99)
**Parity**					
One birth			1		1
Two births			0.91^***^ (0.87-0.96)		1.25^***^ (1.19-1.32)
Three births			0.76^***^ (0.72-0.81)		1.19^***^ (1.11-1.27)
Four or more births			0.59^***^ (0.54-0.64)		0.98 (0.90-1.07)
**Mass media exposure**					
No			0.72^***^ (0.69-0.75)		0.96 (0.91-1.00)
Yes			1		1
**Religion**					
Christianity			1		1
Islam			0.69^***^ (0.65-0.72)		0.58^***^ (0.55-0.618)
Others			0.64^***^ (0.58-0.71)		0.64^***^ (0.58-0.71)
**Wealth index**					
Poorest				0.73^***^ (0.69-0.77)	0.78^***^ (0.74-0.83)
Poorer				1	1
Middle				1.12^***^ (1.05-1.19)	1.10^**^ (1.03-1.17)
Richer				1.22^***^ (1.13-1.31)	1.20^***^ (1.12-1.30)
Richest				1.37^***^ (1.24-1.51)	1.33^***^ (1.20-1.48)
**Sex of head of household**					
Male				1	1
Female				0.91^***^ (0.87-0.96)	0.91^***^ (0.86-0.96)
**Community literacy level**					
Low				1	1
Medium				1.54^***^ (1.47-1.62)	1.21^***^ (1.15-1.28)
High				1.77^***^ (1.65-1.90)	1.21^***^ (1.12-1.31)
**Community socioeconomic level**					
Low				1	1
Medium				1.39^***^ (1.31-1.48)	1.43^***^ (1.34-1.53)
High				1.28^***^ (1.15-1.40)	1.39^***^ (1.28-1.52)
**Place of residence**					
Urban				1.23^***^ (1.15-1.31)	1.18^***^ (1.11-1.26)
Rural				1	1
**Sub-region**					
West Africa				0.69^***^ (0.64-0.70)	1.17^***^ (1.10-1.25)
East Africa				1	1
Central Africa				0.60^***^ (0.56-0.63)	0.69^***^ (0.64-0.73)
South Africa				1.58^***^ (1.38-1.82)	1.28^***^ (1.11-1.47)
**Random effect result**					
PSU variance (95% CI)	0.14 (0.11-0.17)	0.14 (0.12-0.18)	0.20 (0.16-0.25)	0.15 (0.12-0.19)	0.21 (0.16-0.27)
ICC	0.040297	0.0420914	0.0565033	0.044011	0.0601468
LR Test	Chi-square = 281.90, *p* < 0.001	Chi-square = 281.97, *p* < 0.001	Chi-square = 293.22, *p* < 0.001	Chi-square = 244.75, *p* < 0.001	Chi-square = 259.45, *p* < 0.001
Wald chi-square		1339.03^***^	3268.46^***^	2971.7^***^	4802.36^***^
Model fitness					
Log-likelihood	−30,023.153	−29,255.109	−28,252.061	−28,650.877	−27,116.869
AIC	60,050.31	58,516.22	56,532.12	56,803.06	54,289.74
N	52,875	52,875	52,875	52,875	52,875
Number of clusters	1580	1580	1580	1580	1580

Exponentiated coefficients; 95% confidence intervals in brackets

^*^p < 0.05, ^**^p < 0.01, ^***^p < 0.001

## Discussion

The prevalence of early age at first childbirth and SBA among young women in SSA was investigated in this study. We also investigated the relationship between early age at first birth and SBA among young women in SSA. On average, 75.3% of births among young women in SSA were supervised by skilled birth attendants with Chad (38.4%) and Rwanda (93.7%) recording the lowest and highest prevalence respectively. Although the prevalence of 75.3% in SSA was higher than the average of 61% reported between 2014 and 2019 for less developed countries, it was still lower than the average of 99% in developed countries [31]. Again, the prevalence of early age at first childbirth from this study was highest in Chad (85.7%) and lowest in Rwanda (43%). The high prevalence of early age at first birth in Chad could explain why SBA in the country is low. This could be that many of the young women whose first childbirth occurred when they were adolescents may face barriers accessing SBA . Such barriers may include cost of maternal healthcare services, stigma, and negative attitude of healthcare providers [14, 20].

Young women in SSA who had their first birth at the age of 20-24 were 2.7 times more likely to have SBA during delivery than those who had first birth before 20 years. A similar result was reported in 2016 by the Family Health Division of Ghana Health Service, where younger adolescents (10-14 yrs) were less likely to utilize SBA than older adolescents (15-19). In contrast with previous findings in Nigeria [32] and Mali [33] where no statistical significance was found between adolescents’ age and maternal healthcare utilization, our finding supports results that were reported in Pakistan [34]. The possible reason for this finding could be the fear of stigmatization, devaluation, stereotyping, and shaming young pregnant adolescents receive at health facilities [35–37]. In many sub-Saharan African countries, negative social stigma and attitudes towards adolescent pregnancy are deeply rooted in cultural values making it difficult for even some trained health personnel to change them [35, 38, 39]. This makes many young adolescents feel reluctant to access antenatal care and have SBA during delivery. Also, it is reported that women’s autonomy in healthcare decision making increases with age, which affects maternal services utilization [20, 40–43].

Our findings showed that education had a significant relationship with SBA utilization. The lower the education, the less likelihood of SBA utilization and vice versa. Women with no formal education and women who lived in communities with low literacy levels had lower odds of SBA than those with secondary/higher levels of education and includes young women who lived in communities with medium or high literacy levels. This supports previous research output in Nepal [44], Pakistan [35], Namibia [45], Nigeria [46], Gambia [47], and Ethiopia [48–50]. Ameyaw et al. [51] assert that education increases young women’s exposure to information, knowledge, occupation with high income and access to mass media, which subsequently informs their healthcare decisions and utilization of maternal health services. Young women with secondary/higher education levels may be more empowered and informed about the importance of SBA and be more willing to utilize skilled birth delivery services. However, it is argued that community literacy level leads to high uptake and sharing of accurate maternal health information that influences positive maternal healthcare decision-making among adolescents [1].

Our study supports findings of previous studies [52, 53] that showed that women with no mass media exposure had lower odds of SBA during delivery. Mass media, particularly radio and television stations should air health programs to educate people about the pressing health problems in the community. However, those who are exposed to these mass media outlets are more likely to be well informed about SBA services than those with no exposure. Studies have shown that access to mass media education positively affects one’s behavior towards the utilization of health services and SBA [54].

Also, our study found that young women with two or three births in SSA had higher odds of utilizing SBA services compared with their counterparts with one birth. This finding supports previous research conducted in Pakistan and Bangladesh, where women with more than two births were more likely to utilize SBA than women one birth [35, 55]. This could be related with the experience obtained when SBA is utilized for first and subsequent births. For instance, a woman who experienced complications during her first childbirth or had obstetric difficulty may choose to always engage the services of an SBA during subsequent deliveries [56].

Pregnant adolescents residing in urban areas were found to have higher odds of SBA during delivery compared to those in rural areas. This finding highlights the inequalities in access to SBA among young women in SSA and supports other studies in Pakistan [35], Namibia [45], and Ethiopia [48, 57], where young women in urban areas had higher odds of using SBA during delivery.The health facilities in the urban centers might be reasonable for higher utilization of SBA during delivery in SSA. Shorter distance to health facilities in urban centers and better roads and transportation networks, and an increased exposure to mass media and health information may increase the tendency for young women and adolescents to utilize SBA during delivery [48]. In contrast, rural residents might be more influenced by traditional practices.

Furthermore, religion was found to predict the odds of SBA utilization in SSA. Muslim women had lower odds of using SBA during deliveries than Christian women. This corroborates with Ganle’s [58] study findings in Ghana which reported that maternal health services utilization including SBA were low among Muslim women in Northern Ghana. As empirical evidence suggests, religion is a significant predictor of maternal healthcare utilization [59, 60]. There are several factors that could possibly explain why maternal health services utilization among Muslim women is low. One of the reasons could be that religion and culture often interconnect. As argued in the literature, cultural beliefs that affect SBA and maternal healthcare utilization negatively dominate among a religious group [61, 62].

Our findings also show that women in the poorest wealth quintile and women in communities with low socioeconomic levels had lower odds of SBA than women in the middle, richer and richest quintiles and communities with medium to high socio economic status. In contrast, a Nigerian study finding reported no statistical significance between SBA and the socio-economic/wealth quintile of married adolescents [20]. This study's finding, therefore, corroborates with results from a cross-sectional study in Ghana where household wealth was significant in predicting SBA utilization among women [59]. The disparities in the findings could be due to how data was collected on wealth or socioeconomic status in each study setting. This study finding re-emphasizes the need for sub-Saharan African countries to bridge economic inequality that predisposes many disadvantaged adolescents to poor maternal health outcomes, including non-use of SBA.

## Strengths and limitations

One of the strengths of this study is its nationally representativeness. Nationally representative data across 29 sub-Saharan African countries were used. The findings therefore can be generalized to all young women in SSA. Again, data collection techniques and methods used followed best practices and they were used by experienced and well-trained data collectors. This led to a high response rate. Also, the study used advanced statistical models for its analysis in conformance with accepted scientific practices. However, despite these strengths, country-specific findings may not be the same as what has been found across the 29 countries. Again due to the study design, this study cannot generate causal interpretation and the findings and relationships between variables reported from this study may also differ over time.

## Conclusions

Early age at first childbirth has been found to be associated with low skilled assistance during delivery. These findings re-emphasize the need for sub-Saharan African countries to implement programs that will increase the utilization of SBA among young women. We recommend that efforts towards increasing girl child education and ending stigmatization of pregnant adolescents in SSA should be intensified. There is also the need for community sensitisation in the various countries on the effects of adolescent childbearing. Healthcare providers could also educate adolescent girls about contraceptive usage. Further studies should explore the lived experiences of adolescent mothers in accessing SBA in SSA to obtain in-depth information on the challenges adolescents face in accessing SBA services.

## Data Availability

This link provides free access to the data set used in the study: https://dhsprogram.com/data/dataset/

## Abbreviations

AOR: Adjusted Odds Ratio
AIC: Information Criterion
CI: Confidence Interval
DHS: Demographic and Health Survey
SBA: Skilled Birth Attendants
SSA: Sub-Saharan Africa
ICC: Intra-Cluster Correlation
VIF: Variance inflation factor
